# Assessment of Hearing Loss by OAE in Asphyxiated Newborns

**DOI:** 10.5812/ircmj.6812

**Published:** 2014-01-05

**Authors:** Elaheh Amini, Zahra Kasheh Farahani, Mehdi Rafiee Samani, Hamed Hamedi, Ali Zamani, Alireza Karimi Yazdi, Fatemeh Nayeri, Firoozeh Nili, Golnaz Rezaeizadeh

**Affiliations:** 1 Family Health Institute, Breastfeeding Research Center, Tehran University of Medical Sciences, Tehran, IR Iran; 2 Family Health Institute, Maternal, Fetal and Neonatal Research Center, Tehran University of Medical Sciences, Tehran, IR Iran

**Keywords:** Hearing Loss, Otoacoustic Emissions, Spontaneous, Asphyxia

## Abstract

**Background:**

Severe birth asphyxia (apgar < 7 at the 5th minute of birth) is recognized as a hearing loss risk factor by the joint committee on infant hearing (JCIH). About half of the newborns with hearing loss do not indicate any sign and risk factor at birth. Accordingly, the joint committee recommended performance of hearing screening test in 2000, especially for babies born with risk factors.

**Objectives:**

The aim of this study was to evaluate hearing loss in asphyxiated neonates. Early diagnosis would result in early treatment of these newborns.

**Patients and Methods:**

We assessed the relationship between asphyxia and hearing impairment in newborns admitted to a referral hospital, Tehran, Iran within 3 years (2003 - 2006). Hearing problems were diagnosed and followed by otoacoustic emission (OAE) in the third and fifth days of birth. Asphyxiated neonates with abnormal OAE were referred to an ENT specialist; second OAE and tympanometry were carried out after 2 weeks. Based on the results, newborns underwent treatment or were discharged.

**Results:**

Of 149 asphyxiated neonates, 80 had mean first minute apgar score of 4.01, and mean 5th minute score was 7.24. Two percent (3/149) of asphyxiated neonates had abnormal OAEs. No statistical correlation was found between the 5th minute apgar score and abnormal OAE (P value = 0.391). However, a significant relationship between the mean birth weight and abnormal OAE (P value = 0.0406) was found.

**Conclusions:**

It seems that birth asphyxia is not correlated with hearing loss.

## 1. Background

Hearing loss is a common anomaly presenting at birth (1/500 - 1/1000) (1). It is considered that in a representative sample of 10000 neonates, 30 have congenital hearing loss, but only 11 have Down syndrome, five Spina Bifida, and one Phenylketonuria (2). Based on high incidence of hearing impairment, the joint committee in 2000 recommended performance of hearing screening test, especially for high-risk babies (3, 4). Congenital deafness can arise from different causes, including in-utero infection with cytomegalovirus, immaturity, asphyxia, ototoxic drugs, hyperbilirubinemia, and a variety of genetic causes with different modes of transmission. Sensorineural hearing loss (SNHL) in patients with cerebral palsy due to asphyxia has been defined for more than 50 years (5). Adequate oxygenation and perfusion are essential for inner ear function (6) and studies showed that neonatal asphyxia can causes inner ear degeneration, disappearance of the outer and inner hair cells, and degeneration of the spiral and vestibular ganglion cells (7).

Children with impaired hearing, present delays in language learning and general development (8). This problem can only be prevented by early diagnosis and management. Some methods are available for screening of hearing: otoacoustic emission (OAE) and auditory brainstem response (ABR) are two methods of choice for determining/detecting hearing impairment, bedsides being fast, non-invasive, sensitive, and easy to use at neonates, although ABR is more expensive (9). OAE test is generally appropriate for screening neonates’ hearing. Babies who are diagnosed and rehabilitated sooner, demonstrate better language and behavioral skills at the age of five rather than children diagnosed so late (8). Consequently, exorbitant costs of treatment would be prevented. In this study, we have assessed the frequency of neonatal hearing impairment in asphyxiated babies at birth up to 3 years by OAE method.

## 2. Objectives

The aim of this study was to evaluate hearing loss in asphyxiated neonates. Early diagnosis would result in early treatment of these newborns.

## 3. Patients and Methods

Newborn hearing screening by OAE was initiated in Imam Khomeini hospital (Vali-Asr), Tehran University of Medical Sciences (TUMS), as a neonatal referral and academic center, (Tehran, Iran) (2003 - 2006). The target population included every asphyxiated newborn with first minute Apgar score < 5, and/or 5th minute Apgar score < 6. This case-series study was approved as a student thesis by the research and medical ethics committee of TUMS to the Helsinki declaration. Some variables of mothers and babies were collected in questionnaires (maternal age, education, type of delivery, drug usage, past history, neonatal weight, gestational age, Apgar scores, hypoxic delivery, hyperbilirubinemia, infectious disease history, and meconium staining).

OAE screening was performed by Echo Screen TE machine (Madsen Electronic, Copenhagen, Denmark) that uses transient-evoked otoacoustic emission. At the end of the test, the result was shown on the screen as “PASS” when there was OAE response (response to the stimulus at > 35 dBHL (decibels Hearing Level)), and REFER when there was no response to the stimulus (9). Screening test was performed between the 3rd and 5th days of birth. Babies with a normal OAE were discharged, but in cases with abnormal OAE (uni or bilateral ), after referring to a pediatric Ear, Nose and Throat (ENT) specialists (specialized in child hearing loss) for diagnosis confirmation and management, second OAE and tympanometry were performed after 2 weeks. In cases with liquid or negative pressure, treatment was recommended, and then OAE and tympanometry were performed once more. In cases with abnormal OAE and normal tympanometry, ABR was performed immediately. If the baby had first abnormal OAE and second normal OAE, final OAE was performed in the 3rd month. In cases with hearing loss after determination of threshold, early intervention (hearing aids and rehabilitation) and audiologic follow up by behavioral tests were recommended. Data were analyzed by SPSS statistical package (version 13). P < 0.05 was indicated as significant.

## 4. Results

In this study 149 newborns with birth asphyxia were evaluated for hearing loss. Demographic and clinical characteristics of the asphyxiated neonates are shown in Table 1. Of 149 cases, 38 (25.5%) failed the test (13 unilateral, 25 bilateral). Among them, only 22 neonates came for the 2nd OAE, 13 of which had abnormal OAEs (6 unilateral and 9 bilateral), and finally 6 children went through the third step of screening process, three of which had abnormal test results. Of 149 asphyxiated neonates, 40 were born with NVD, 10 (25%) failed the test, and of 109 cases (73.2%) with C/S, 28 cases (25.7%) failed the test (P value = 0.031). Of 38 cases who failed the test, 22 were male and 16 were female, who failed the TEOAE (P value = 0.914).

**Table 1.** General Characteristics of Asphyxiated Neonates

	Number: 149
**Age, mean ± SD, week**	35.5 ± 3.36
**Sex, No. (%)**	
Female	60 (40)
Male	89 (59)
**Weight, mean ± SD, gr**	2363 ± 899
**Apgar (1st min)**	4.01
**Apgar (5th min)**	7.24
**Type of delivery, No. (%)**	
C.S	109/73 %
NVD	40/26 %
**Total**	149/100 %

The highest and lowest gestational ages were reported 40 and 26 weeks, respectively. Newborns with abnormal tests represented mean gestational age of 34.12 ± 3.7 weeks and mean birth weight of 2014.21 ± 344 g compared with those with normal OAEs with mean gestational age of 36.05 ± 3.7 weeks and mean birth weight of 2551.35 ± 446 g (P value = 0.0406). Of 149 cases, 80 had first minute Apgar score of less than 5, 28 of which (35%) failed the test (mean Apgar score of first minute was 4.01), but among these neonates only 24 had 5th minute Apgar score of less than 6 (mean Apgar score = 7.24), and 5 (22.7%) failed the test. But in 69 cases, first minute and 5th minute Apgar scores were higher than 5 and 6 respectively, asphyxia diagnosis based on clinical signs of the first hours of birth, fetal bradycardia, and meconium stained amniotic fluid. It is noteworthy that no significant relationship was found between abnormal OAE and 5th minute Apgar score of less than 6 (P value = 0.391).

## 5. Discussion

Hearing loss can be considered as one of the most important birth defects. Birth asphyxia and ischemia have often been thought to be major causes of early hearing loss or deafness. Experiments have confirmed/shown that hypoxia induces the ABR elevation threshold in rat and cat neonates (10). Several studies have confirmed that the incidence of hearing loss among babies in NICU with low Apgar scores in the first and 5th minutes of birth is much higher than the general population (2 - 4%). They can have a high rate of middle ear pathology, which would potentially affect their OAEs (11). Early detection of hearing loss especially in high-risk babies by screening at, or shortly after birth, and appropriate interventions, are critical to speech, language and cognitive developments. In addition, Tower in his study emphasized that ABR has to be done for these babies firstly; after failing this test, OAE should be the next step (12). However he didn’t find any significant differences between the sample and control group. He stated that influential high-risk factors such as Asphyxia, hyperbilirubinemia, low birth weight, mechanical ventilation, familial history of hearing loss, and hospital stay in NICU more than 2 days, should be considered. Each neonate might be evaluated seriously regarding hearing impairment.

Of 149 neonates in our study, approximately 2% (3/149) had abnormal OAEs, but we may underestimate this rate, since some of our cases with abnormal OAEs did not continue the follow up. Our finding was more than the incidence of hearing loss at birth (1/500 - 1/1000), however, in another study the rate of abnormal OAE at high-risk newborns was reported approximately 2% (13). Furthermore, Ohl and his colleagues in 2009 found that 3 - 5% of at–risk neonates suffer from hearing loss (8). Another study from Saudi Arabia reported this rate of prevalence about 1.3% (14). It seems that in our complicated neonates, large number of causative factors might be involved affecting the incidence of hearing loss. In our study, a positive relationship was found between the mean birth weight and normal OAE. Presumably, abnormal OAE with low mean birth weight could be due to presence of IUGR and low birth weight (LBW) babies. Previous studies showed that the incidence of hearing impairment in premature and LBW babies is 20 times more than babies with normal weight. Two percent of newborns with < 1500 g suffered from hearing loss (15, 16).

Babies who were born by C/S had more abnormal OAE in comparison with NVD-born babies (P value = 0.031). It seems that spinal or epidural anesthesia had some effects on the hearing system (7). In addition, it is noteworthy that these babies were probably at a higher risk of emergency delivery due to issues such as prematurity, PROM, and LBW. Olusanya et al. in 2004 reported some significant risk factors for hearing impairment such as young maternal age, prolonged and obstructed labor, prematurity, and prolonged rupture of membranes. These factors also have a great role in the type of delivery (14). Twenty-two males (24.7%) and 16 females (26.7%) failed the TEOAE (P value = 0.914) in our study. Therefore, we could not see any important difference between the two genders, although almost all investigations support that hearing loss is more common in males than females. Male to female ratio of 1.2:1 was reported (17); boys may be at high risk for hearing loss, since they are more prone to serious neonatal diseases such as RDS and sepsis (15). One study mentioned high frequency of unilateral hearing loss in boys and equally-distributed bilateral hearing loss between males and females (18).

We also found that there is no significant correlation between congenital hearing loss and exclusively the 5th mean Apgar score as an important feature for severity of asphyxia. This finding is compatible with former studies, too (5). However, some studies have pointed out that asphyxia and low Apgar score are the reasons for temporary hearing loss, not permanent status (19). Moreover, in another study carried out in Shanghay hospital, Japan, the effects of prolonged asphyxia during parturition on auditory brain stem were assessed. They did not find any tremendous impact of asphyxia on this neural part (20). Furthermore, Jiang et al. in 2004 reported that after 3 days hypoxic-ischemic damage to central auditory system, it tends toward recovery and after 1 week the system recovers significantly (20). Another study in 1999 indicated that fetal hypoxia, of any degree and duration, is not a particular reason for permanent hearing loss (10). Finally, Bergman in 1984 confirmed that Apgar scores, low PaO2 and high PaCo2 were not independent risk factors for hearing loss (15). Familial history of hearing loss could be one major cause of abnormal OAE (2), but we didn’t study this factor in our investigation.

As a matter of fact, evaluation of the exact etiology of neonatal hearing loss in children with complicated deliveries is difficult because of the large number of causative factors involved. However, it seems that birth asphyxia is not correlated with hearing loss; at the same time it is impossible to say that the relative importance of different factors and their interactions are clearly identified. Indeed, given the results of newborns hearing loss screening in majority of countries, we are no longer in doubt that hearing screening should be performed for all newborns, especially the ones at risk during neonatal period.
